# Simple Optical Fiber Sensor for Express and Cross-Sensitive Hydrogen Detection

**DOI:** 10.1021/acssensors.5c04316

**Published:** 2026-01-07

**Authors:** Elena Miliutina, Yuliia Viktosenko, Andrii Trelin, Vasilii Burtsev, Vladislav Buravets, Tomas Hrbek, Vaclav Svorcik, Oleksiy Lyutakov

**Affiliations:** † Department of Solid State Engineering, 52735 University of Chemistry and Technology, 166 28 Prague, Czech Republic; ‡ Materials Centre, Faculty of Science, J. E. Purkyně University, Pasteurova 3544/1, 400 96 Ústí nad Labem, Czech Republic; § Department of Surface and Plasma Science, Faculty of Mathematics and Physics, Charles University, V Holešovičkách 2, 180 00 Prague, Czech Republic

**Keywords:** plasmon, optical fiber, hydrogen detection, Pd, PDMS

## Abstract

The utilization of hydrogen as an energy source is becoming more and more widespread. Since hydrogen is a highly explosive gas, its use requires the development of inexpensive and simple sensors capable of measuring hydrogen concentrations under a variety of conditions. These sensors must meet several parameters, such as small size, light weight, corrosion resistance, and remote operation capability. The ideal hydrogen sensors should also be insensitive to the presence of various interfering gases and humidity or temperature variation and be protected against potential poisoning. In this work, we present a simple optical hydrogen sensor that satisfies most of the above criteria. The sensor is based on a plasmon-active multimode optical fiber coated with Pd and PDM layers in a stepwise manner. The Pd layer, deposited on the plasmon active area, ensures sensitivity toward hydrogen through hydrogenation of Pd, leading to a significant shift in the plasmon absorption band wavelength position. An additional PDMS layer ensures sensor protection against various interfering gases (NO_2_, CH_4_, CO_2_, CO, and NH_3_), including the moisture of sulfur-containing compounds. The sensor response is measured within tens of seconds, while its regeneration takes approximately 2 min. The operating temperature range is from RT to 80 °C, with a slight decrease in sensor functionality at an elevated temperature. The proposed structure is simple, allows the removal of hydrogen detection, and can be used under various operation conditions.

## Graphical abstract

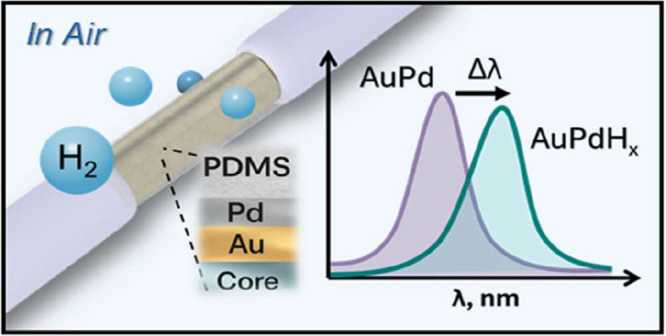

Hydrogen is widely recognized as a clean energy source capable of replacing fossil fuels, reducing in this way the CO_2_ emissions and cutting down the negative impact of human activity on the environment.^1–3^ However, the widespread use, transport, and storage of hydrogen will give rise to a range of challenges and safety risks, as this gas is highly flammable, easily ignited, colorless, and odorless.^4–6^ To avoid explosive accidents and maintain the use of hydrogen at an acceptable risk level, the development of reliable, fast, sensitive, and selective hydrogen sensors is one of the main tasks of hydrogen technology. Traditional sensors are based on catalytic, electrochemical, mechanical, optical, acoustic, or resistance-based detection and commonly include two or more electrodes in their design.^7–13^ Such sensors cannot withstand electromagnetic interference and, moreover, pose a risk of hydrogen explosion due to the random generation of electrical sparks.

In contrast, optically based hydrogen sensors do not have these shortcomings. In particular, fiber-optic sensors provide additional advantages such as small size, light weight, corrosion resistance, and remote operation capability.^14–18^ Such sensors can be easily installed and used in various external environments, where the presence of hydrogen should be monitored, including small reaction chambers, electrochemical cells, underground conditions, or liquid environments.^19–22^ Most fiber hydrogen sensors use palladium- or tungsten-oxide-sensitive layers, which can change their optical properties due to interaction with hydrogen.^10,23–26^ In the case of Pd-based sensors, hydrogen is dissociated from the metal surface under normal conditions (RT and atmospheric pressure), and the created hydrogen atoms easily diffuse through Pd with the production of palladium hydride.^27^ This conversion causes a change in the refractive index of the material and produces significant volumetric expansion, leading to an alteration in the transmitted optical signals.^14,15,27–30^ The related changes are subsequently read by using various optical concepts, including the use of the plasmon resonance effect, interferometry, and the grating-related phase shift of the probe light beam.^29,31–35^

On the other hand, Pd suffers from lower stability under real conditions because its surface can be easily poisoned or oxidized, with a loss of functionality. The oxidation of Pd results in the formation of a thin oxide film, which acts as a barrier to hydrogen, slowing its dissociation and decreasing sensor functionality in terms of sensitivity and response speed.^35^ In addition, the high level of external humidity interferes with the sensor response, resulting in detection inaccuracy.^28,36,37^ To overcome these issues, the addition of various selective membranes (mainly polymer-based or compounds such as MOFs were also reported^38–42^ ) was proposed as the cladding of the Pd-based sensitive optical fiber area.^40,41^ Small hydrogen molecules can diffuse through the membrane and react with a sensitive Pd layer.^38^ Simultaneously, the addition of the membrane prevents direct contact with alternative poisoning molecules, such as CO, H_2_O, or sulfur-containing contaminants, making the sensor highly sensitive toward a small concentration of hydrogen molecules.

Inspired by previous studies, we propose a simple sensor design, based on common multimode optical fibers with a plasmon-active area covered by Pd and an additional polysiloxane-based membrane. In our design, the presence of the Au layer allows one to excite an apparent plasmon absorption band, the intensity and wavelength position of which are sensitive to the state of the surrounding medium (i.e., Pd or palladium hydride), while the polymer layer ensures selectivity toward hydrogen and protects Pd from poisoning. We investigated the sensor functionality under various experimental conditions and demonstrated its selectivity toward hydrogen in relatively severe experimental conditions.

## Results and Discussion

### Main Experimental Concept and Sample Optimization

The particular steps of hydrogen sensor preparation are presented in Figure 1. First, the naked fiber core was obtained by thermally removing the polymer shell. On the fiber core, a thin layer of Au was deposited, ensuring the excitation of the surface plasmon under light transmission through the fiber (Figure S1). Importantly, the surface plasmon manifested itself only when the fiber was surrounded by a medium with a suitable refractive index (Figure S2). In particular, the light absorption measurements, performed in air, demonstrate the absence of the plasmon at visible wavelength with the use of Au- or Au@Pd-covered fibers, while after the deposition of an additional PDMS layer (with a refractive index equal to ≈1.4), the apparent plasmon absorption band appears.

**Figure 1:**
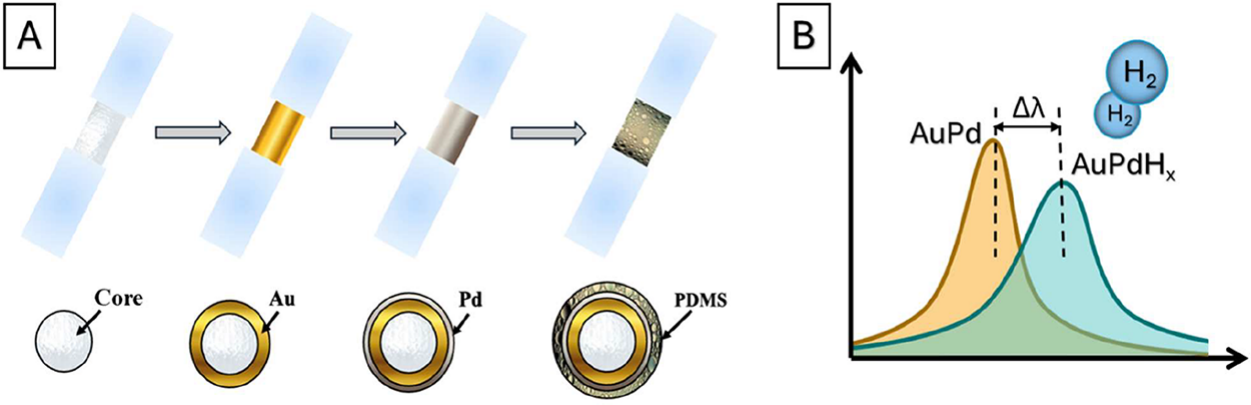
(A) Schematic representation of hydrogen sensor preparation, including the creation of the plasmon active area on the fiber core, deposition of a hydrogen-sensitive Pd layer, and a hydrogen-selective PDMS layer. (B) Proposed excitation of the surface plasmon band in the transmitted light and its shift due to Pd hydrogenation.

In turn, the use of alternative polymers with a higher refractive index (PMMA with *n* ≈ 1.49 or PS with *n* ≈ 1.59) did not result in the appearance of a plasmon absorption band at visible wavelengths (Figure S3). Alternatively, the use of a fluorinated acrylate polymer coating with a lower refractive index (*n* ≈ 1.37) also allows measuring the position of the plasmon absorption band located in the 600–850 wavelength range (Figure S4). However, in this case, an additional step (UV curing) was necessary, and we focus mainly on a more “simple” PDMS. As mentioned above, the proposed fiber-optic sensor is based on probing the changes of Pd optical parameters by a plasmonic evanescent wave. The changes occur due to Pd hydrogenation and related changes in optical constants and volumetric expansion (Figure 1B). The thin layer of PDMS ensures selectivity toward hydrogen, protects the sensor against poisoning, and reduces the emergence of false-positive signals.

It should also be noted that we used the previously optimized thickness of the Au layer, which corresponds to ca. 2100 nm/RIU sensor sensitivity. The thickness of the Pd layer was additionally optimized, using the deposition of various amounts of Pd and subsequent measurements of the plasmon band in underwater conditions (Figure S5). The addition of Pd layers results in a gradual shift of the plasmon absorption band and its widening (the peak widening can result in a decrease in sensor quality). After the optimization, a thickness of Pd equal to ∼5 nm was chosen as a compromise value between the amounts of deposited Pd and the ability to read the wavelength position of the plasmon absorption band.

### Characterization of the Sensor Composition and Structure

The surface morphology and composition of the hydrogen optical fiber sensor created are shown in Figure 2. First, SEM measurements indicate the relatively smooth surface of the optical fiber, while EDX analysis reveals the presence of the characteristic Au, Pd, and Si signals. In addition, EDX mapping confirms the homogeneous distribution of both plasmon-active (Au) and hydrogen-selective Pd across the optical fiber surface (Figures 2 and S6). Additional AFM measurements confirm the apparent changes in the surface morphology after the deposition of Au and Pd layers (Figures S7–S9), accompanied by an increase in surface roughness. The surface morphology also changes significantly after the deposition of the PDMS layer, which completely screens the cluster-like morphology, characteristic of sputtered metal(s) (Figure 2B vs Figures S8 and S9). The scratch tests, performed after the deposition of metals and PDMS, reveal the thickness of particular layers, which were found to be ca. 20 nm for Au, 5 nm for Pd, and ca. 170 nm for cladding the PDMS layer (Figure S10). It should be noted that we used the smallest thickness possible of the homogeneous PDMS layer (determined by the speed of fiber rotation during the PDMS curing, which was found to be 210 rpm). An additional increase of the rotation speed results in the creation of a nonhomogeneous PDMS coating, leaving areas of Pd unprotected by the polymer (Figure S11). It was also possible to create a thicker PDMS layer using a lower rotation speed, but the sensor performance worsened in this case (discussed below). It is also worth noting that PDMS is not the only polymer that can be used for hydrogen detection in the proposed sensor design. Replacement of PDMS with acrylate also allowed hydrogen detection (Figure S12). In turn, the Raman measurements of the final sensor structure indicate the appearance of 2906, 2965, and 488 cm^–1^ peaks, which correspond to symmetric and asymmetric stretching vibrations of the −CH_3_ groups and Si–O–Si backbone bending vibrations of the PDMS (Figure 2C and Table S1). Subsequently, Raman mapping performed using two bands, located at 2906 and 2965 cm^–1^, after the fiber rotation, indicates the homogeneous distribution of the PDMS layer across the fiber surface (evident as a homogeneous intensity of Raman signal—Figure 2E).

**Figure 2:**
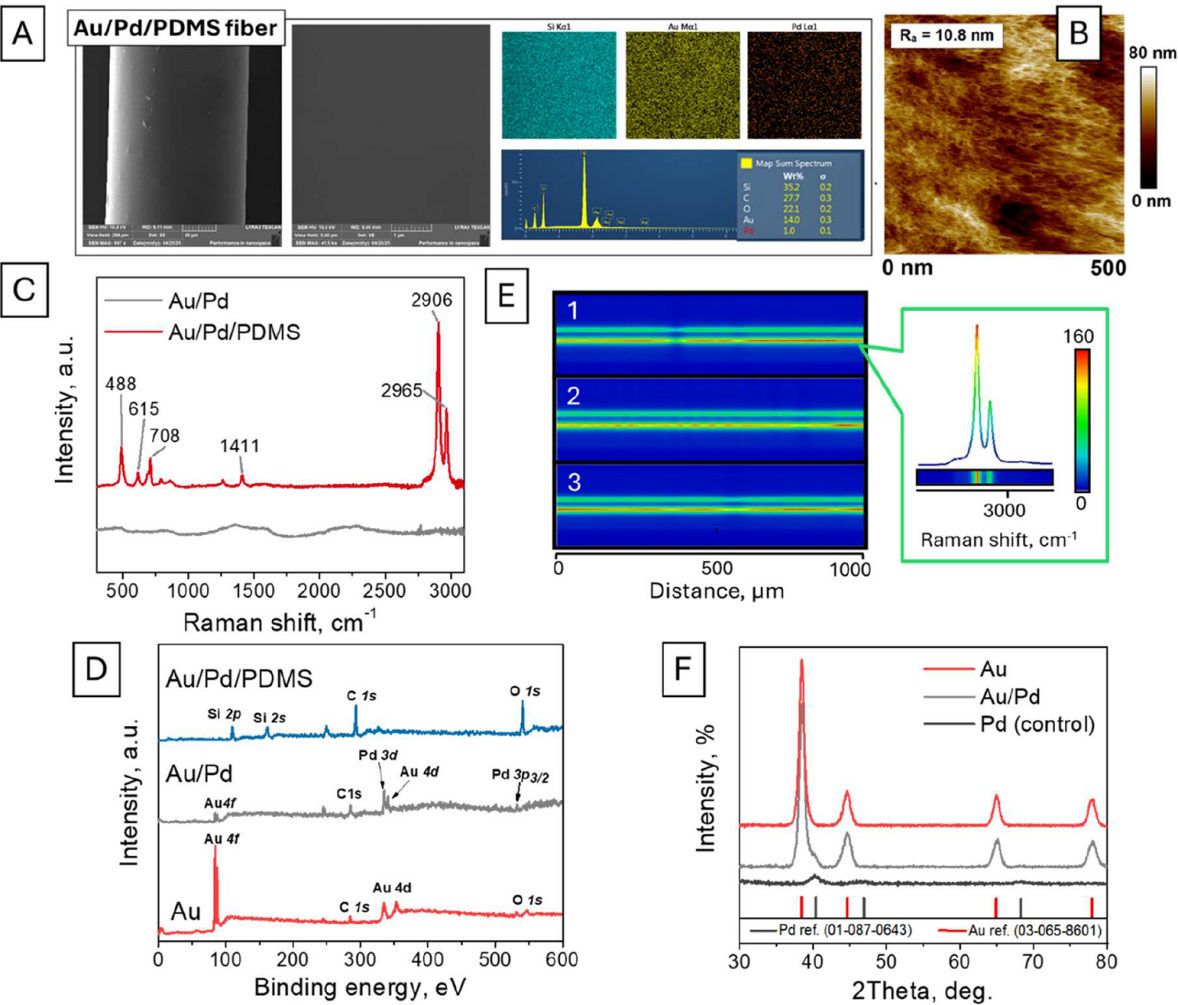
(A, B) SEM-EDX and AFM-based characterization of the Au@Pd@PDMS fiber surface. (C) Raman spectrum of PDMS on the fiber surface. (D) Several line-based Raman maps along the fiber surface, measured along the fiber after its rotation at 0°, 90°, and 180° angles. (E) High-resolution XPS scan of Pd. (F) XRD pattern measured in the glazing angle mode on the surface of several closely packed Au fibers and Au@Pd fibers.

We also performed XRD and XPS characterization of the Pd-sensitive layer (before the deposition of PDMS). The XPS results reveal the appearance of Au peaks, after fiber coating with gold, which are subsequently screened after Pd addition. In turn, the deposition of the PDMS layer results in the appearance of Si, C, and O peaks and the complete disappearance of characteristic signals from Au and Pd, which can be expected because of the higher polymer thickness. The XPS results also reveal that palladium is deposited in the Pd(0) state (Figure S13), ensuring the excellent reactivity toward hydrogen (unlike Pd oxide, where the declaration of hydrogenation kinetics was reported previously). In turn, the XRD pattern (Figure 2F) of the Au-coated fibers reveals the presence of several reflexes, corresponding to the polycrystalline Au layer. After the addition of the Pd layer, the gold reflexes were also observed, but their slight shift and broadening indicate the presence of a thin layer of palladium in a metal form. Importantly, no reflexes from Pd oxide or carbide were observed, which can be formed as a result of Pd contamination during deposition and worsen the sensor functionality.

### Sensor Functionality in Hydrogen Detection

In the next step, the functionality of the created fiber sensor was tested (Figure 3A). First, the optical response to hydrogen presence was estimated as a shift in the plasmon absorption band position that occurred because of the hydrogen present in the air (at RT and normal pressure). A shift of the plasmon absorption band by about 4 nm occurs at a hydrogen concentration of 5 vol % vol. An increase in the hydrogen content results in more pronounced changes in the plasmon absorption band position, which reaches the 30 nm value at the 40 vol % H_2_ concentration. The calibration curves depicting the position of the plasmon absorption band maximum vs hydrogen concentration are presented in Figure 3B. As is evident, the sensor shows an almost linear response in the 0–40 vol % concentration range. Subsequent increase in the hydrogen concentration leads to a sensor saturation, probably due to complete hydrogenation of the available Pd surface.

**Figure 3:**
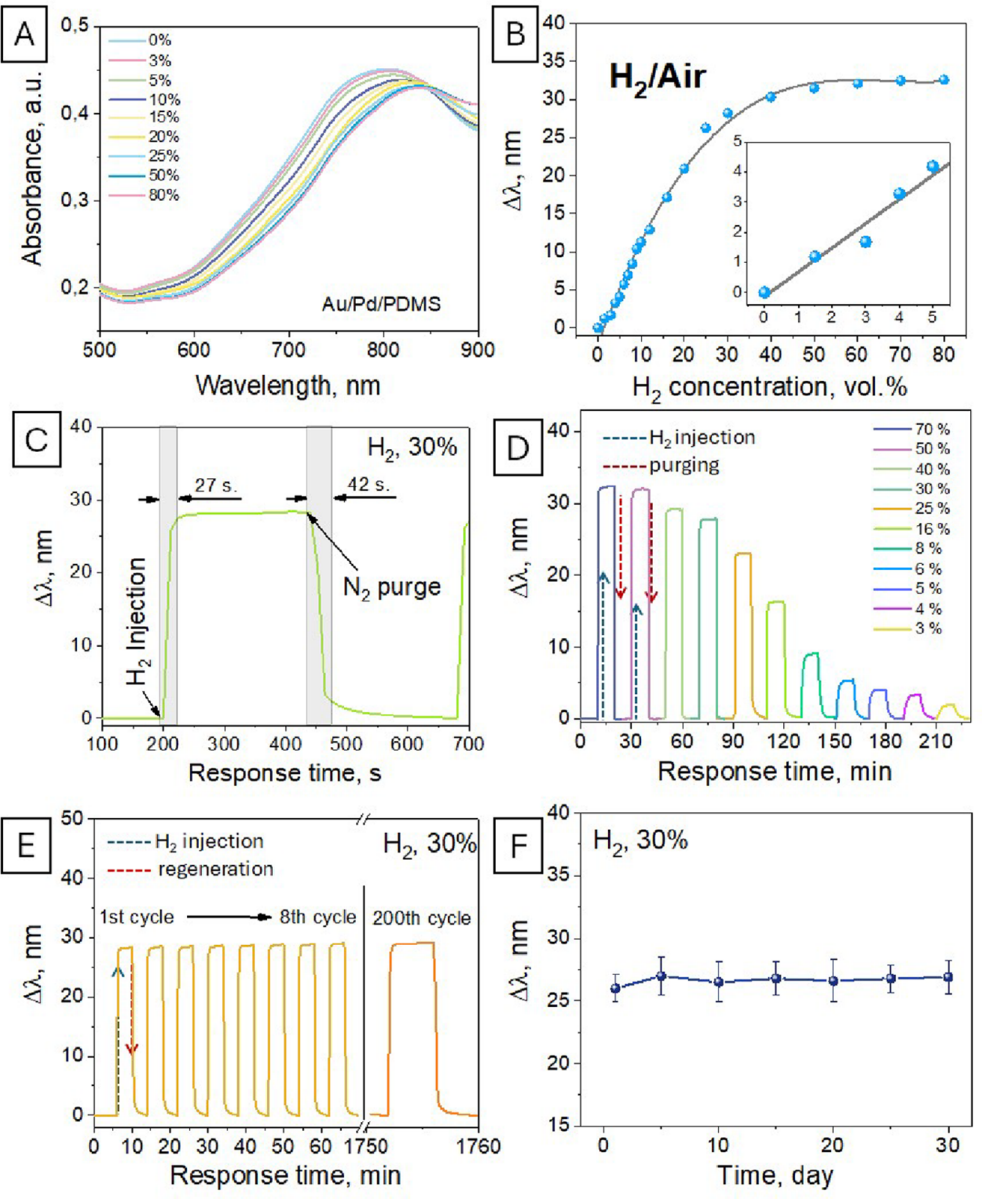
(A) UV–vis absorption curves, measured in the transmitted light mode with the use of the Au@Pd@PDMS fiber sensor in a mixture of hydrogen with air. (B) Calibration curves revealed the position of the plasmon absorption band maximum as a function of the hydrogen concentration in air; the inset shows the sensor response on the lower hydrogen concentrations. (C) Time-resolved response of the sensors. (D–F) Demonstration of functionality with the use of different hydrogen concentrations (D), long-term operation (E), and storage under ambient conditions (F).

In turn, the time-resolved response toward the presence of hydrogen is shown in Figure 3C as a single cycle of hydrogen addition and subsequent sensor regeneration under purging with ambient air. As is evident, the optical response (evident as a start of plasmon absorption band shift) of the sensor occurs with an ca. 5 s delay after the hydrogen injection. The maximal shift is reached during ca. 30 s. Sensor regeneration takes a longer time, and the plasmon absorption band returns to the initial value after ca. 45 s. It should also be noted that the use of a thicker PDMS layer resulted in the apparent deceleration of both sensor response and regeneration times (Figure S14). The functionality of the sensor was additionally determined using several cycles of hydrogen treatment, regeneration, and subsequent utilization with a gradually decreasing H_2_ concentration (Figure 3D). As is evident, the fiber sensor then returned to the initial value of the plasmon absorption band position in a relatively short time, and the lower concentration of hydrogen can be detected independent of the previous exposure to a higher concentration(s). The long-term sensor operation is illustrated in Figure 3E, where 200 subsequent hydrogen detection cycles are presented. In this case, the sensor was exposed to hydrogen for relatively long time intervals (30 h), subsequently regenerated by air purging, and used again. Perfect result reparability was reached in this case, despite repetitive use of the sensor. No changes in the sensor response were observed, indicating the good stability of the created structure. Finally, Figure 3F reveals the sensor stability after the storage at ambient conditions. In particular, sensor response measurements were performed every 5 days for 1 month of storage under ambient laboratory conditions, and no differences in the sensor response were observed, confirming long-term sensor utilization ability.

### Sensor Functionality for Hydrogen Detection in Close-to-Real Conditions

In the next step, the sensor functionality was tested under close-to-real conditions, considering the impact of potential temperature and humidity variations as well as the presence of alternative gases. First, the impact of temperature was estimated to be in the 25–80 °C range (Figure 4A), and the results are presented as calibration curves. An increase in temperature results in the negligible shift in the optical fiber response in the 25–40 °C range. The subsequent temperature rise results in a more pronounced but still low decrease in sensor sensitivity, evident as a less pronounced shift of the plasmon absorption band position. Probably, such a phenomenon should be attributed to a decrease in the hydrogen solubility in Pd at elevated temperatures. It should also be noted that higher temperatures significantly accelerate the sensor response and shorten its regeneration time (Figure S15).

**Figure 4:**
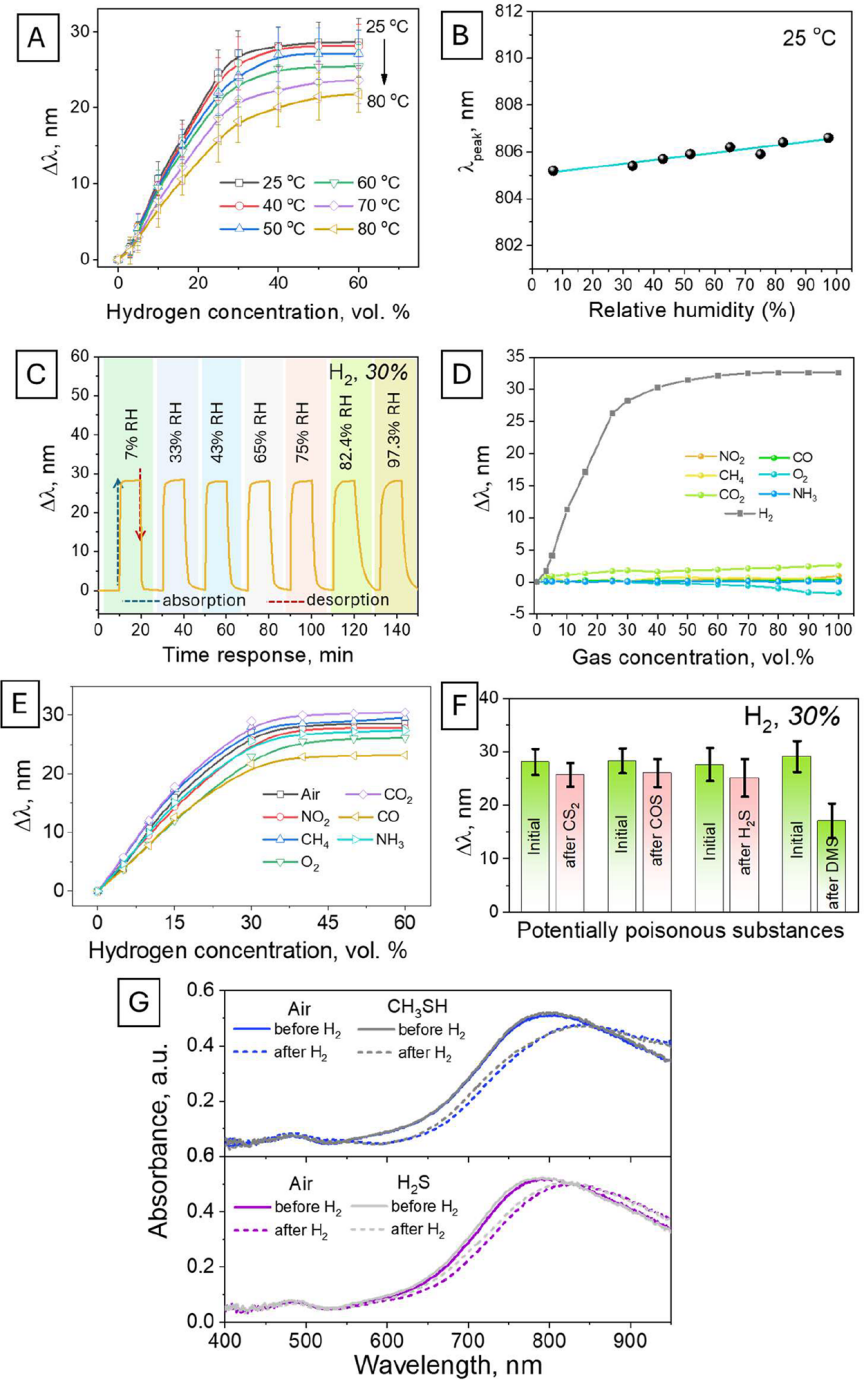
(A) Sensor response to different hydrogen concentrations, measured at different temperatures. (B, C) Position of the maximum plasmon absorption band at different humidity levels in the air and results of hydrogen detection in the air saturated with moisture. (D) Position of the maximum plasmon absorption band at different concentrations of alternative gases in the air. (E) Calibration curves of the sensor response measured during hydrogen detection in its mix with different gases. (F) Sensor response, measured before and after its interaction with potential sulfur contaminants. (G) UV–vis spectra reveal the position of the plasmon absorption band, measured before and after sensor treatment with H_2_S and CH_3_SH vapor.

The impact of humidity was verified by exposure of the sensor to air saturated with water vapor or a hydrogen/air mixture under increased humidity (experiments were performed at RT). The position of the plasmon absorption maximum, measured in air, was found to be insensitive to the presence of water molecules (Figure 4B). In particular, no changes in the sensor response toward hydrogen presence were observed up to 100% humidity level (Figure 4C). The absence of humidity-induced false responses, commonly observed for Pd-based hydrogen sensors, should be attributed to the hydrophobic nature of PDMS (Figure S16), which prevents the surface sorption and diffusion of water molecules toward the sensitive Pd layer. Therefore, the created sensor is humidity-insensitive and, thus, satisfies one of the more important requirements for real hydrogen sensing.

The sensor response toward the presence of alternating gases was also measured, and the obtained dependencies are presented in Figure 4D,E. In the case of the addition of NO_2_, CH_4_, CO_2_, CO, and NH_3_, the plasmon resonance position was conserved up to 100% vol. concentration of these gases in the air (Figure 4D), indicating perfect selectivity toward hydrogen and absence of the interfering contribution to sensor response. Only the addition of oxygen negatively affects the sensor response. Even some “opposite” shift of the plasmon absorption band position was observed at a higher oxygen concentration. Generally, the observed absence of interference from alternative gases can be attributed to their limited diffusion through the homogeneous PDMS layer due to the relatively higher molecules size as well as the gas polarity.

Next, we performed a range of cross-sensitivity experiments aimed at hydrogen detection under different backgrounds. For this goal, different concentrations of hydrogen (10–60% vol. range) mixed with different gases were measured (Figures S17–S22). The main results are presented in Figure 4E, and as expected, the optical fiber response was almost insensitive to the presence of NO_2_, CH_4_, CO, and NH_3_, and a similar shift of the plasmon absorption band was observed independently in the hydrogen mixing with these gases or “inert” air. Only in the case of oxygen and CO presence, a decrease in the sensor response was indicated. This phenomenon can be attributed to the enhanced diffusion of these gases (compared to other gases) through the PDMS layer at its higher partial pressure. In this case, the gas molecules are able to reach the Pd surface and produce the metal oxide layer in the case of oxygen (or block the available Pd sites in the case of carbon monoxide), which worsens the sensor response.

We also investigated the sensor stability against potential poisoning by sulfur-containing molecules. These experiments were carried out in the two modes: using fiber immersion in a solution of potentially poisoning molecules and sensor interaction with H_2_S and CH_3_SH vapor. In the first case, the optical fiber sensor response was initially measured; then, the sensor was immersed in a solution containing potential sources of sulfur poisoning (such as H_2_S (water solution, ca. 3.9 g/L concentration), CS_2_, carbonyl sulfide (COS), and dimethyl sulfide (DMS)), dried in air, and the sensor response to the presence of hydrogen was measured again. The results obtained, presented in Figure 4F, indicate that a similar shift in the plasmon absorption band was measured before and after the sensor interaction with sulfur-containing contaminants (except for the DMS case). Therefore, the presence of a PDMS layer efficiently protects the Pd from most sulfur contamination. In the case of DMS, an apparent swelling of DMS is observed, and even in this case, the poisoning of Pd only worsens the sensor functionality. Even better results were observed in the case of sensor interaction with H_2_S and CH_3_SH vapor (Figure 4G). The sensor response was not affected by the previous interaction of the sensor with these potentially poisoning molecules. It should be noted that H_2_S is a common contaminant in the case of natural gas, while mercaptan(s) are commonly added to the gas during its transport and delivery. The insensitivity of the created sensor to these common contaminants makes it possible to detect hydrogen content in the case of production, transportation, and technological use of natural gas. Generally, we can conclude that the proposed optical fiber design allows the measurement of hydrogen without the risk of Pd surface poisoning by commonly present sulfur-containing molecules, making hydrogen detection possible under relatively complex and close-to-real conditions.

We also compare our results with an alternative optical fiber sensor used for hydrogen detection. The comparative results are presented in Table 1, and as is evident, the majority of the hydrogen sensors are focused on the detection of extremely low hydrogen concentrations.^43–60^ Only a few attempts have been made to achieve hydrogen detection against the background of interfering compounds or with a stable sensor background. In our experimental arrangement, the detection of hydrogen at the parts per million level is difficult to achieve. However, the selectivity of our sensor significantly outperformed those of previously reported ones. In turn, the speed of sensor response and regeneration is at a level comparable to those of the results previously published. An additional advantage of the proposed sensor is its simplicity of preparation (simple metal sputtering and polymer deposition), usage (only common light sources and spectral analyzer are required), and stability. It should also be mentioned that the proposed sensor design is rather aimed at the detection of higher hydrogen concentrations (detection of smaller concentrations is also possible (see Figure 3B inset), but additional parameters such as external temperature should be taken into account in this case). Thus, the optical sensor is rather aimed at the use in devices such as bioreactors, electrochemical cells, or gas pipelines, where higher hydrogen concentrations should be detected and small optical fibers can be introduced simply, with minimal space required and minimal disruption of system integrity. In addition, changes in the experimental setup (for example, the use of a narrow band light source and the detection of transmitted light intensity) will potentially allow even the detection of a smaller hydrogen concentration, below the hydrogen explosive limit.

**Table 1:** Comparison of the Optical H_2_ Sensor Performance, Proposed in This Work, with Previously Published Results

type	sensing layer	detection range, %	response//recovery time, s	source
optical fiber with Bragg grating	Au/Pd	0.17–1.02	37//49	^43^
fiber-optic sensor	PdNPs in PMMA matrix	0.1–5	25//90	^44^
optical fiber with Bragg grating	PdCl_2_/AuNPs	0.2–1.2		^45^
shaped fiber with Bragg grating	Pd NPs (8 nm)	1–5	90 s/140 s	^46^
shaped optical fiber	Pd–Au alloy	0.25–10	30//	^47^
flat sensor, transmission measurements	ordered Pd array	100 (low H_2_ pressure)	100//-	^48^
flat substrate, colorimetric detector	Pd-capped Y thin film	0.1–3	10//	^49^
flat sensor, transmission measurements	Ta–Pd alloy with PTFE layer (30 nm)	0.1, 4, 100%	<1//120	^50^
plasmonic nanowire	Pd–Ag alloy	2.5–3.6	2//∼8	^51^
plasmonic nanoarray	Au@Pd NPs	4 (background insensitive)	few minutes	^52^
fiber coupled to Fabry–Perot resonator	Graph./PdNPs	0.02–3	18//-	^53^
flexible, flat substrate. Transmission measurements	PdNPs/Teflon/PMMA	5 (CO insensitive)	8//12	^54^
optical fiber with coated tip	PdNPs in Teflon	4 (low pressure)	2.5//6–8	^55^
optical fiber with Bragg grating	WO_3_–Pd	1–8	40//120	^56^
plasmon-active optical fiber	Au/Pd	0.5–4	10//15	^57^
fiber coupled to Fabry–Perot resonator	Pd thin film	0.5–3.5	10//25	^58^
optical fiber with Bragg grating	Pd thin film	0.05–0.4	4//20	^59^
optical fiber with Bragg grating	Au–Pd thin film	0–0.7	26//40	^60^

## Conclusions

In this work, the simple design of a plasmon-active optical fiber sensor, able to ensure remote hydrogen detection at various operating conditions, is presented. As a sensor basis, a plasmon-active optical fiber, covered with Pd and PDM layers, was used. The presence of a plasmon absorption band allows one to measure the optical signal in the light transmission mode. The hydrogenation of Pd layers is responsible for the apparent shift of the plasmon absorption band wavelength position, ensuring the detection of hydrogen, while the PDMS layer provides selectivity toward hydrogen. A linear and quick sensor response (on the order of tens of seconds) was measured in the 0–40 vol % hydrogen concentration range, while the saturation of sensor response was observed at higher concentrations. The high selectivity of the created structure toward hydrogen was also demonstrated, and no interfering or negative impact of gases such as NO_2_, CH_4_, CO_2_, CO, or NH_3_ was observed. Only higher oxygen partial pressures were found to slightly decrease the sensor response. Successful hydrogen detection was also demonstrated independent of the humidity level, but it was slightly dependent on temperature variation in the RT–80 °C range. The stability of sensor response and no changes of the response toward hydrogen were demonstrated during 200 operation cycles performed for 20 h; moreover, the sensor storage at ambient conditions for 1 month does not affect its functionality. Finally, the addition of a PDMS layer efficiently protects the sensor against sulfur contamination and related Pd poisoning. The proposed sensor design is simple and robust, and the created structure can be easily used in various operating conditions where the presence of hydrogen as a result of leakage should be monitored with a high reliability level.

## Experimental Section

### Materials

Multimode plastic-clad silica (PCS) optical fibers were purchased from CeramOptec (Germany) with core and buffer/cladding diameters of 200 and 230/500 μm, respectively. Au and Pd sputtering targets (Au 99.99%, Pd 99.99%) were obtained from Safina (Czech Republic). The Sylgard 184 Silicone Elastomer Kit was purchased from Dow (USA). Polymethylmethacrylate (PMMA) and polystyrene (PS) were provided by Goodfellow (UK). The EFiRON prepolymer (PC-409 LV, RI 1,37) was supplied by Fospia. Acetone (p.a.), ethanol (p.a.), deionized water, and chloroform (p.a. ≥ 99.5%) were purchased from Sigma-Aldrich.

### Sample Preparation

A centimeter of PCS fiber shell was thermally removed; the naked cylindrical surface of the silica fiber core was purified by washing it with deionized water, acetone, and ethanol and dried in a desiccator. Thin films of Au and Pd were deposited on the PCS fiber core by vacuum sputtering. The deposition of both metals was performed under continuous rotating fiber. The deposition of polydimethylsiloxane (PDMS) was carried out from the corresponding Sylgard kit, by mixing the particular components, immersing the optical fiber in the fresh prepolymer solution immediately after mixing, pulling it out, and drying the PDMS core with constant rotation of the fiber (Figure S23). The curing of PDMS was performed in an ambient temperature (24–27 °C range) for 12 h. For the creation of different PDMS thicknesses, the fiber rotation speed was varied from 10 to 1500 rpm.

### Measurement Techniques

Atomic force microscopy (AFM) was performed in scan-assist mode using an Icon microscope (Bruker, Germany). The AFM scratch test was carried out on the fiber core by profiling across a premade scratch. Scanning electron microscopy (SEM) and energy-dispersive X-ray spectroscopy (EDX) were carried out using a LYRA3 GMU microscope (TESCAN, CZ) operated at an accelerating voltage of 10 kV.XPS spectra were obtained using an EnviroESCA device (SPECS) fitted with a monochromated Al Kα X-ray source working at 1484.71 eV. Concentrations of elements were calculated in at. % using the manufacturer’s sensitivity factors. X-ray diffraction (XRD) patterns were obtained using an XRDynamic 500 diffractometer (Anton Paar, Austria) with a Cu Kα X-ray source. The diffraction data were processed by using Panalytical HighScore Plus 4.0 software. Raman spectra were collected with 785 nm laser excitation wavelength, using a DXR3 Raman Microscope (Thermo Fisher Scientific, USA). The water contact angle (CA) was measured on a Drop Shape Analyzer DSA100 (Krüss, Germany) at room temperature.

### Measurements of Sensor Response

A gas chamber with the ability to control the composition of the gas environment was used to study the response of fiber-optic sensors. The experiments were carried out in the light transmission mode. The optical fiber sensor was connected to an AvaLight-DHS light source (Avantes, The Netherlands) and an HR2000+ (Ocean Optics, USA) spectrometer in the 200–1100 nm wavelength range using fiber-optic couplers supplied by Thorlabs. Hydrogen absorption was carried out by supplying H_2_/air (or alternative gases) mixtures with a hydrogen concentration ranging from 1 to 80% vol. Desorption tests were carried out by replacing the gas environment with pure air.

Unless otherwise stated, hydrogen detection experiments were performed at room temperature and zero humidity. The active part of the sensor was placed in a homemade chamber with a volume of 25 mL through two hermetically sealed holes. Hydrogen (as well as other gases or their mixtures with hydrogen) was purged through the chamber at an initial rate of 15 mL/s, which ensured complete renewal of the chamber volume in a few seconds. Then, the purging rate was reduced to 2 mL/s to maintain a constant atmosphere inside the chamber.

In the case of experiments at elevated humidity, the gas mixture was saturated with water vapor from solutions of inorganic salts (amount produced: CaCl_2_ – 7.0%, MgCl_2_ – 33.0%, K_2_CO_3_ – 43.0%, NH_4_NO_3_ – 65.0%, NaCl – 75.0%, KCl – 82.4%, K_2_SO_4_ – 97.3%). A commercial humidity sensor (DHT22, Waveshare Electronics) was used for control measurements of the humidity level. The temperature-dependent experiments were performed in a Binder MK56 climate chamber.

Methanethiol vapors were produced in situ by the addition of HCl to a solution of sodium thiomethoxide in water and bubbling with Ar. The created vapors of the produced methanethiol were directed in the chamber with a previously placed hydrogen fiber sensor. The sensor was treated with H_2_S vapor in a similar way—bubbling of the hydrogen sulfide solution with Ar and the subsequent interaction of the created H_2_S vapor with the Au/Pd/PDMS surface.

## Supplementary Material



## Data Availability

All supporting information (data) has been deposited in Zenodo at https://zenodo.org/records/17964712.

## Notes

Plasmon absorption bands, measured on Au, Au/Pd, Au/Pd/PDMS, Au/Pd/PS, and Au/Pd/PMMA-coated fibers; optimization of Pd thickness; SEM-EDX and AFM characterization of the pristine fiber, and Au- and Au@Pd-coated fiber surface; AFM scratch tests revealed the thickness of particular layers in the Au@Pd@PDMS structure; high-resolution XPS of the Au and Pd characteristic region; sensor response and regeneration time as a function of temperature; water contact angle measurements; affiliation of the Raman bands; image of the experimental setup used for PDMS curing; SEM image of the PDMS coating prepared using an increased rotation speed; sensor response as a function of the PDMS thickness: light absorption spectra and sensor response measured using Au@Pd@EFiRON; sensor response to hydrogen in a mixture of NO_2_, O_2_, CO_2_, CO_,_ NH_3_, and CH_4_ (PDF).
