# Supplementary material for: Simple Optical Fiber Sensor for Express and Cross-Sensitive Hydrogen Detection
Source: ACS Sens. 2026 Jan 7;11(1):756–65. doi: 10.1021/acssensors.5c04316 (PMC12836353; doi:10.1021/acssensors.5c04316)
Supplement: Supplementary file 1 [file se5c04316_si_001.pdf]

Supporting Information for:

Simple Optical Fiber Sensor for Express and Cross-Sensitive Hydrogen Detection

Elena Miliutina[a,b*], Yuliia Viktosenko[a], Andrii Trelin[a], Vasilii Burtsev[a], Vladislav Buravets[a], Tomas Hrbek[c], Vaclav Svorcik[a], Oleksiy Lyutakov[a]

[a]*Department of Solid State Engineering, University of Chemistry and Technology, 16628 Prague, Czech Republic*

[b]*Materials Centre, Faculty of Science, J. E. Purkyně University, Pasteurova 3544/1, 400 96, Ústí nad Labem, Czech Republic*

[c]*Department of Surface and Plasma Science, Faculty of Mathematics and Physics, Charles University, V Holešovičkách 2, 180 00, Prague 8, Czech Republic*

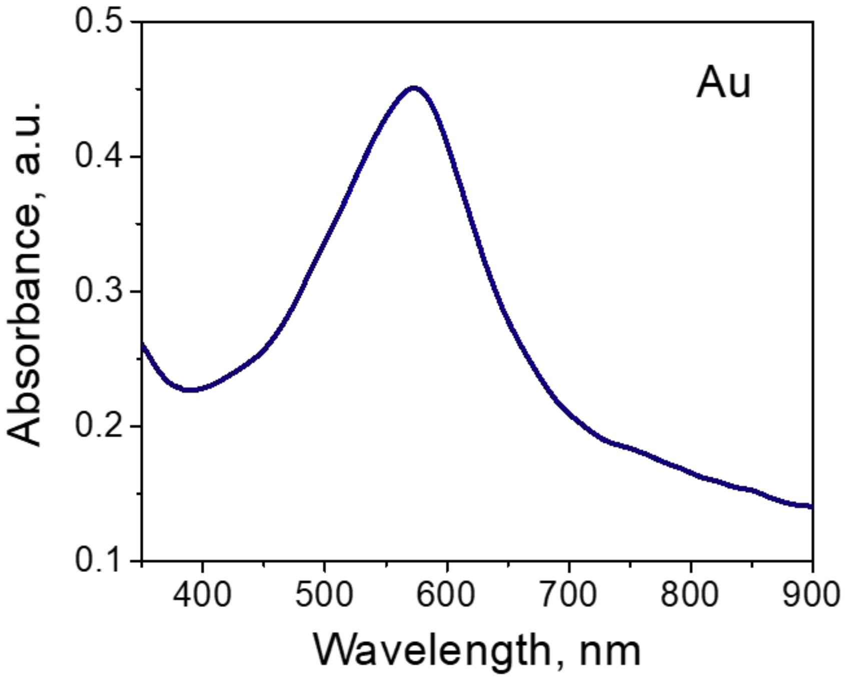

Fig. S1 Plasmon absorption band, measured on Au coated fibre in the light transmission mode (measurements were performed under underwater conditions).

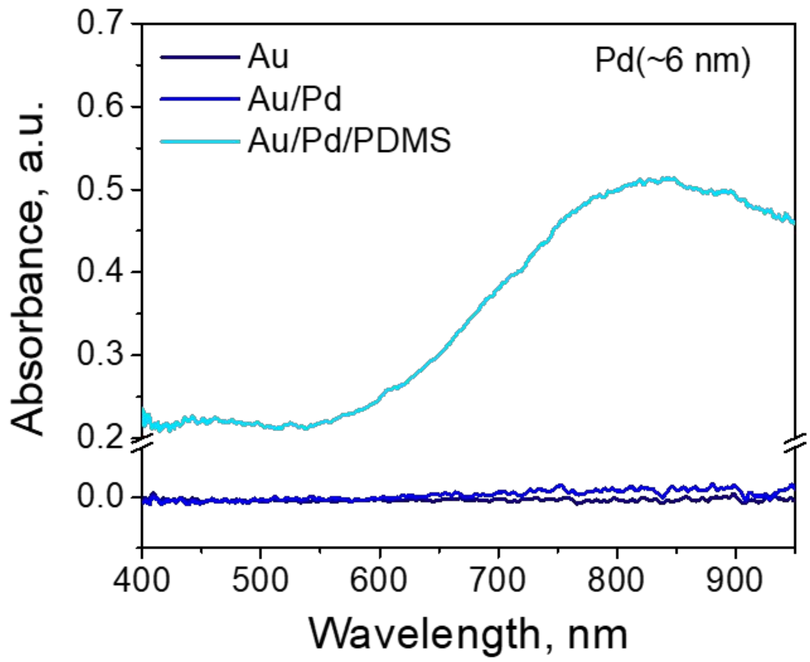

Fig. S2 Light absorption spectra measured with the use of Au, Au@Pd and Au@Pd@PDMS coated fibre: The appearance of the plasmon absorption band is evident only after the addition of PDMS layer.

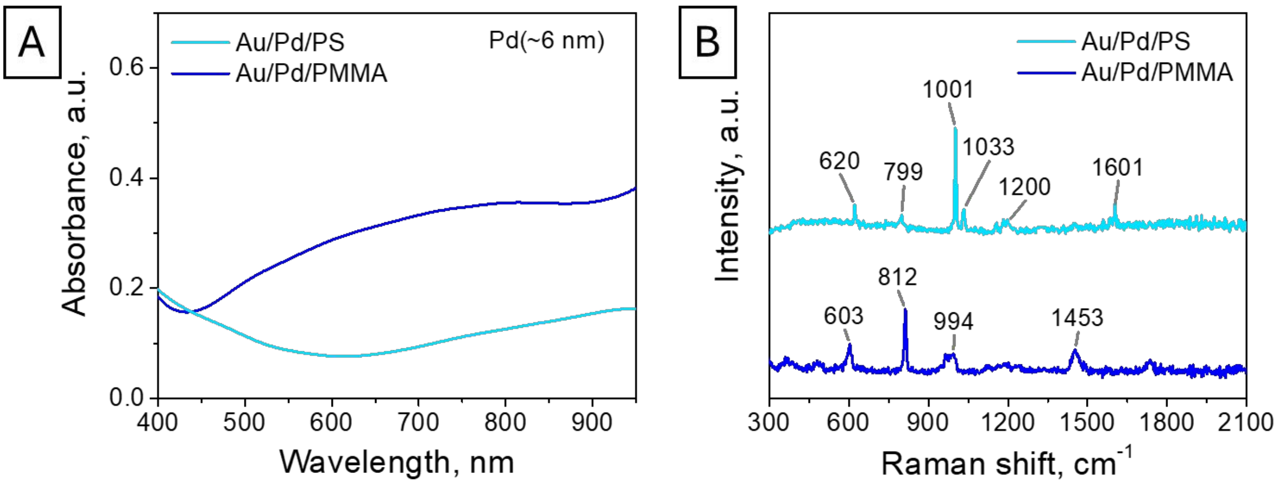

Fig. S3 Utilisation of alternative polymers with a higher refractive index (PMMA and PS): (A) - absence of apparent plasmon absorption band at Vis-NIR wavelengths is evident; (B) – Raman measurement confirmed the presence of PMMA and PS on the fibre surface.

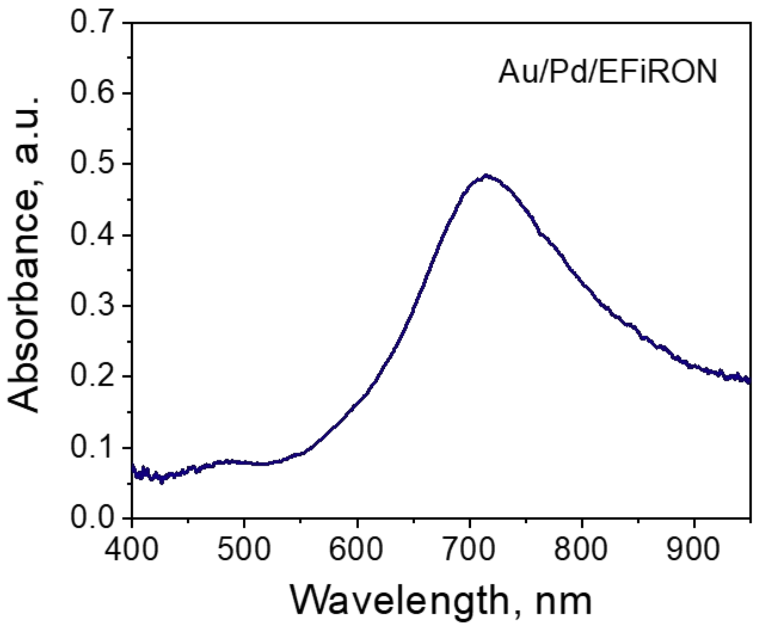

Fig. S4 Light absorption spectra measured with the use of Au@Pd@EFiRON (fluoribnated acrylate, PC-370, RI 1.37) coated fibre: the appearance of the plasmon absorption band is evident after the fibre is coated with an alternative polymer, with low refractive index.

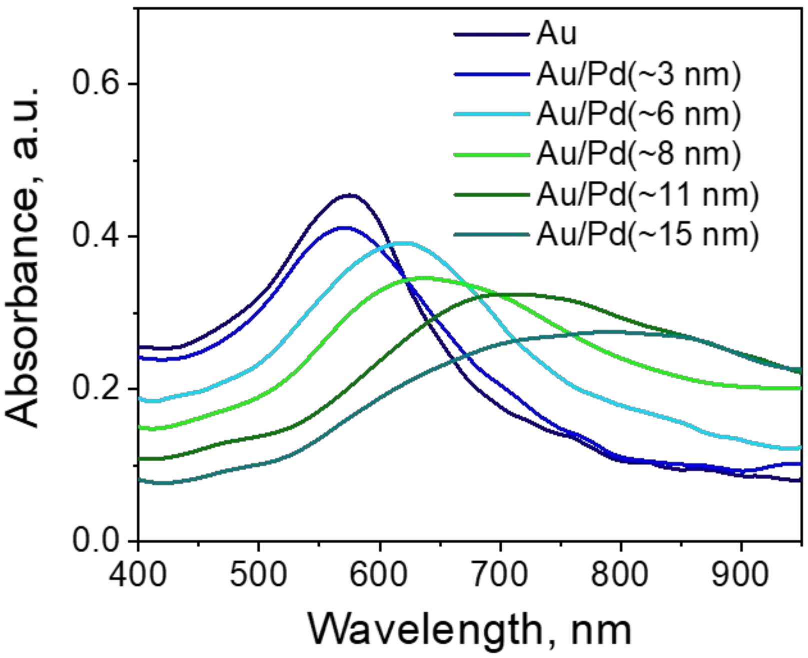

Fig. S5 Optimisation of Pd thickness: measurement of the position and width of the plasmon absorption band after deposition of the Pd layer with various thicknesses on the Au layer (measurements were made without the addition of the PDMS layer at underwater conditions).

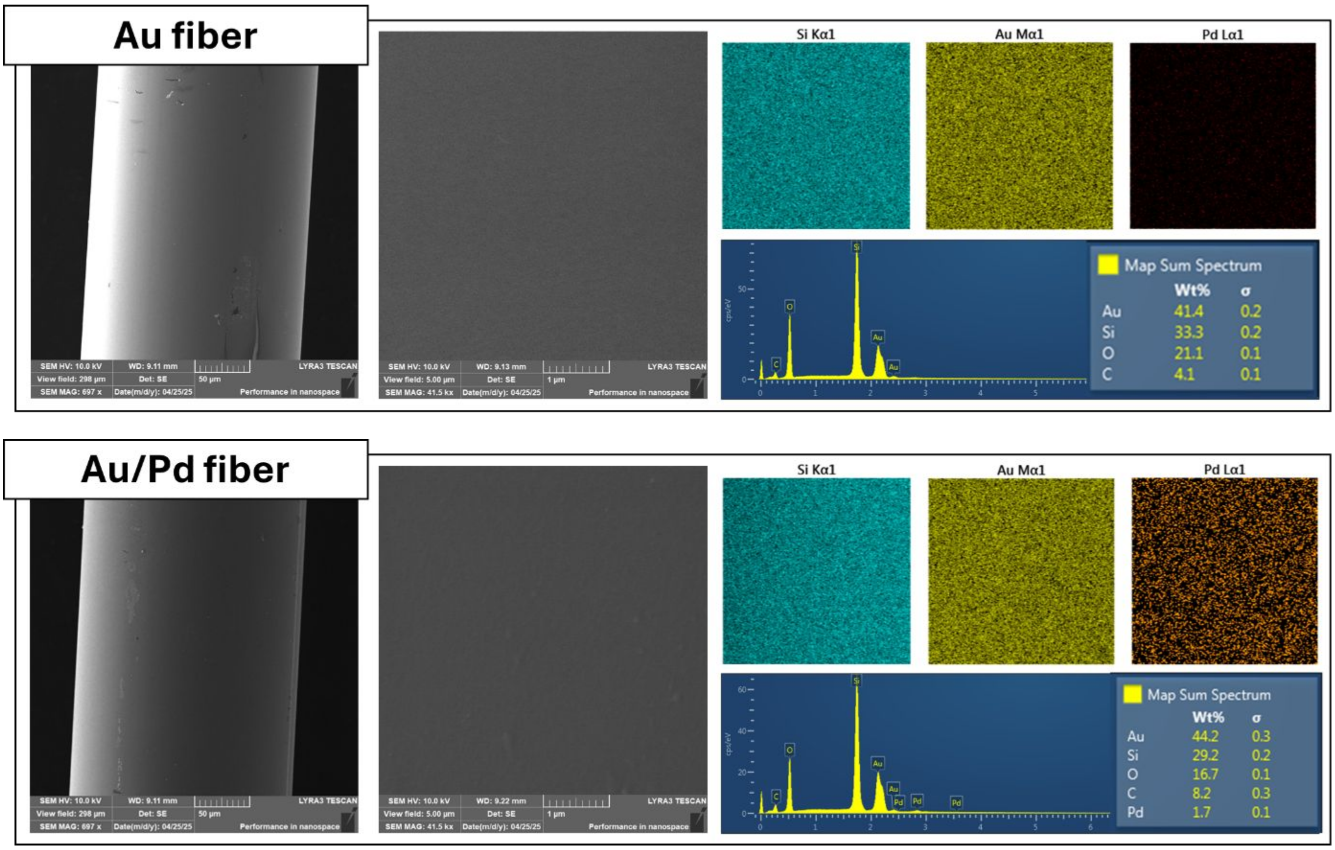

Fig. S6 SEM-EDX characterisation of the Au and Au@Pd fibre surface.

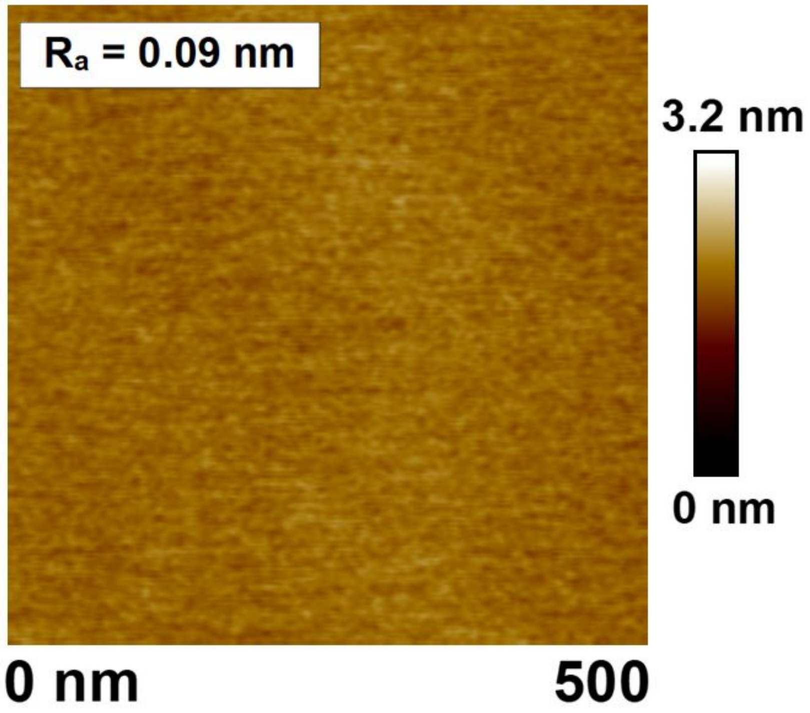

Fig. S7 AFM measured surface morphology of the pristine naked optical fibre core.

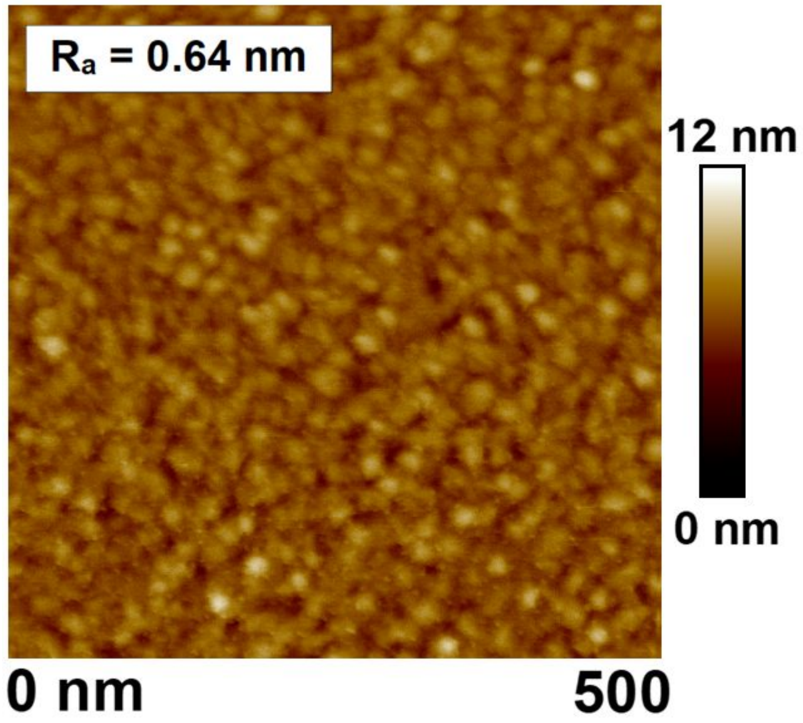

Fig. S8 AFM measured morphology of Au coated optical fibre.

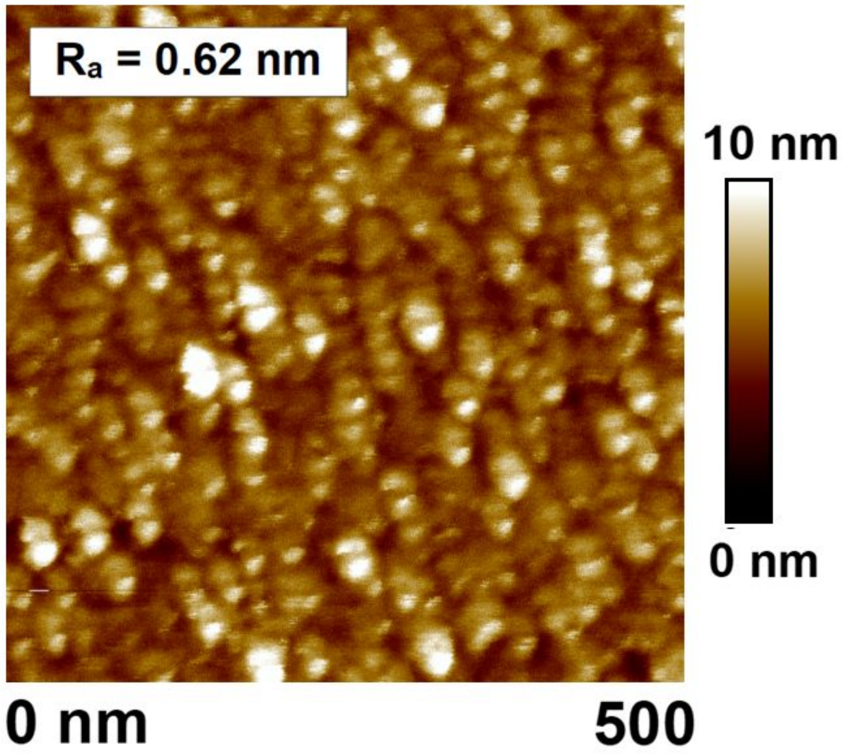

Fig. S9 AFM measured morphology of Au@Pd coated optical fibre.

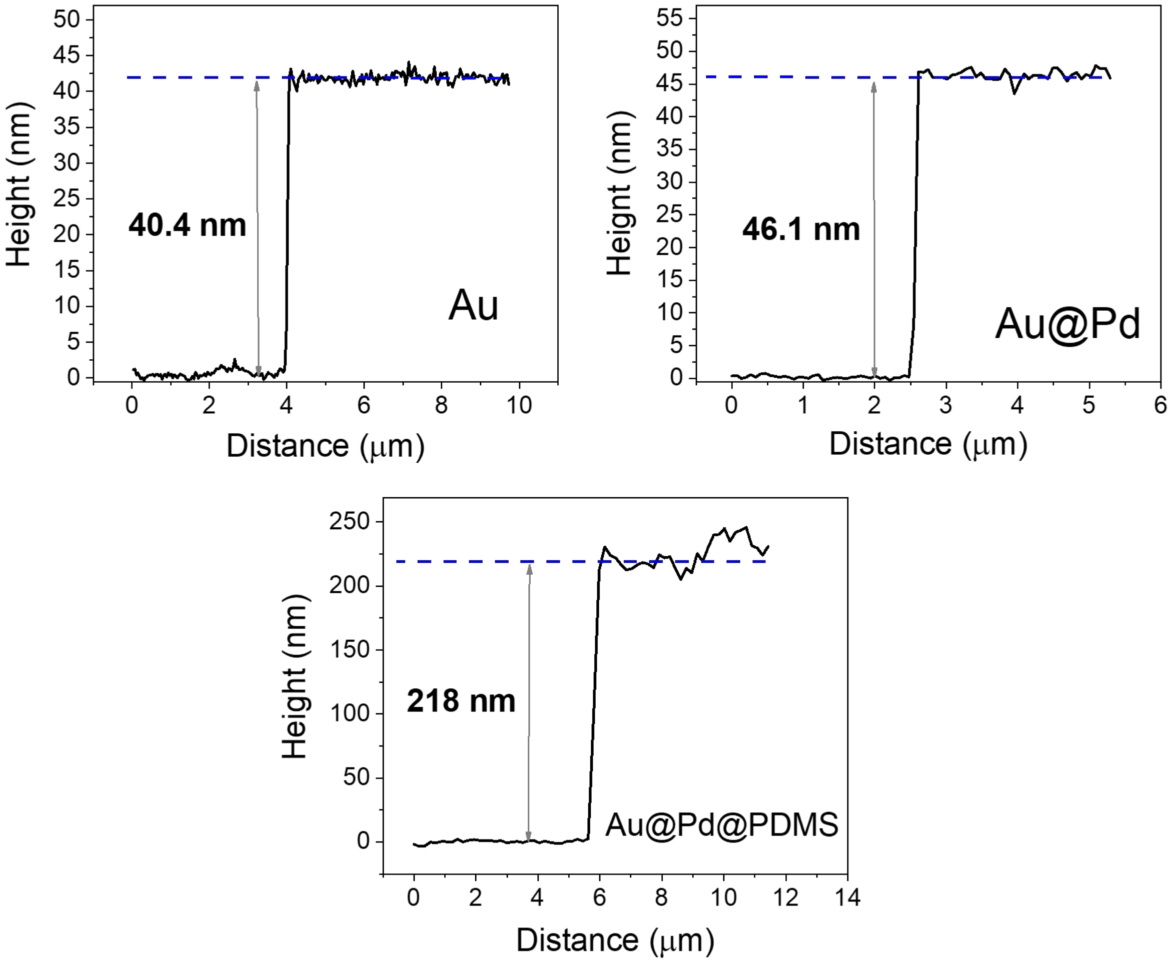

Fig. S10 AFM scrat tests performed for the thickness determination of particular layers in Au@Pd@PDMS structure: Au thickness is equal to 40.4 nm; Pd thickness is equal to 5.7 nm, PDMS thickness is equal to ca. 178 nm.

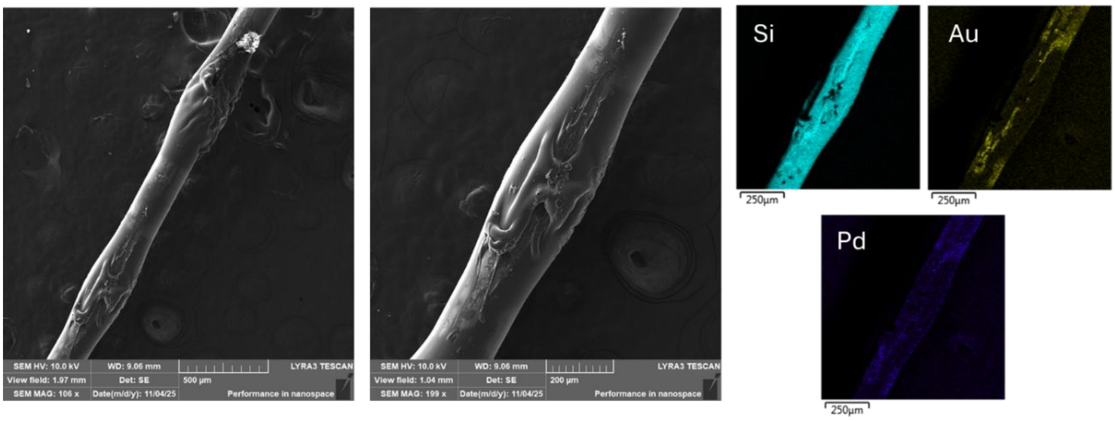

Fig. S11 SEM image of the PDMS coating, prepared using an increased rotation speed (which can result in the PDMS coating with lower thickness but led to the creation of a non-homogeneous PDMS layer).

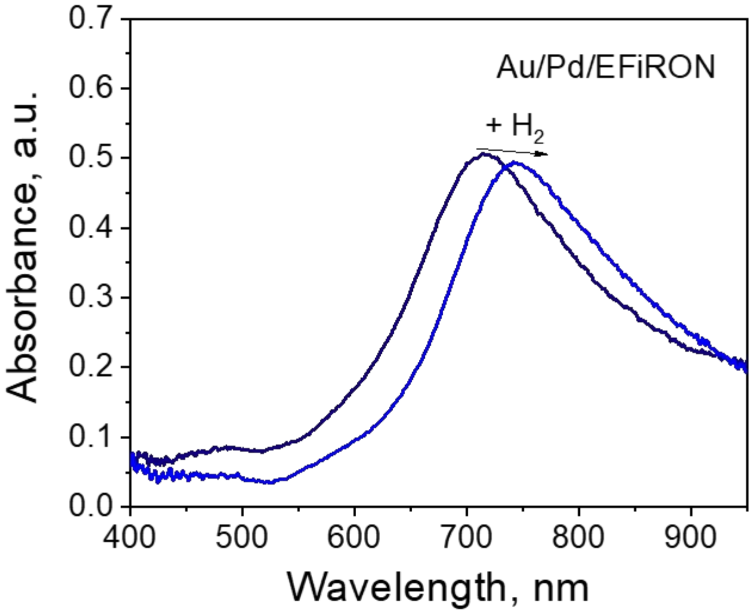

Fig. S12 (A) – UV-Vis absorption curves, measured in transmitted light mode with the use of Au@Pd@EFiRON fibre sensor (50 % vol. of H_2 in the air).

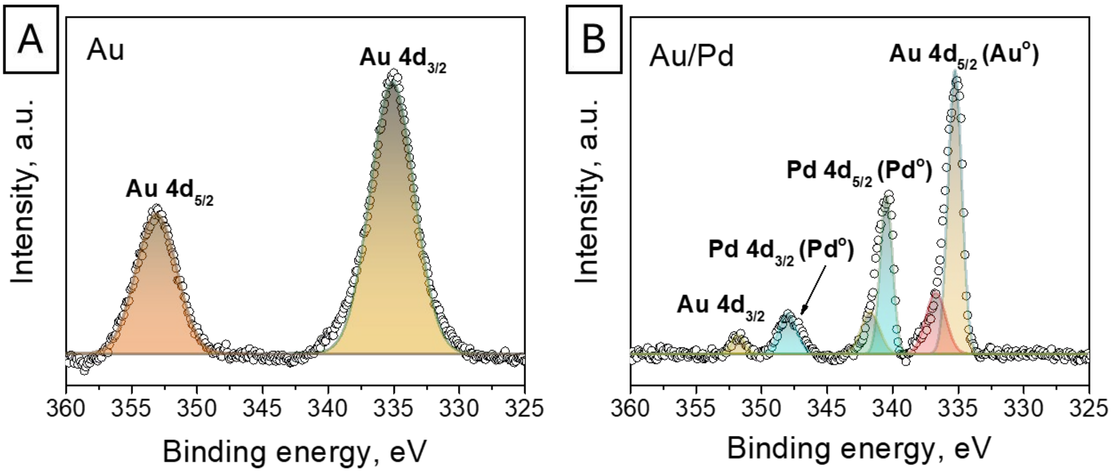

C

Sample	Surface atom element concentrations (at %)				
	C *1s*	Pd *3d*	O *1s*	Au *4f*	Si *2p*
Au	53.0	0.0	5.8	34.0	7.2
Au/Pd	62.9	18.8	13.7	4.6	-
Au/Pd/PDMS	46.6	0.4	29.9	0.0	23.1

Fig. S13 Additional XPS results: high resolution of the Au and Pd characteristic region and surface atom elements concentration (at. %).

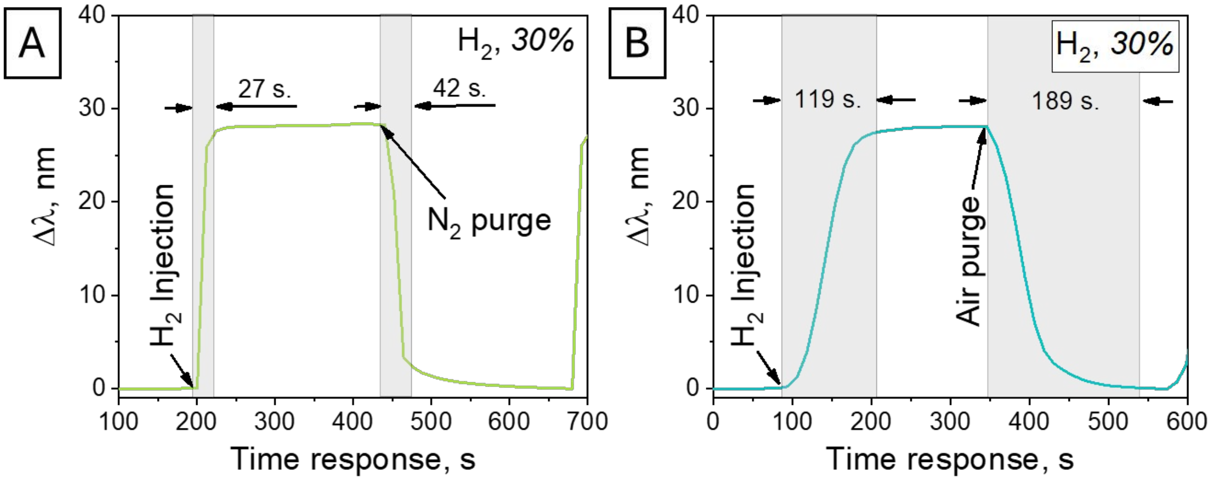

Fig. S14 Sensor response and regeneration time as a function of PDMS thickness: A – 170 nm and B – 2 200 nm.

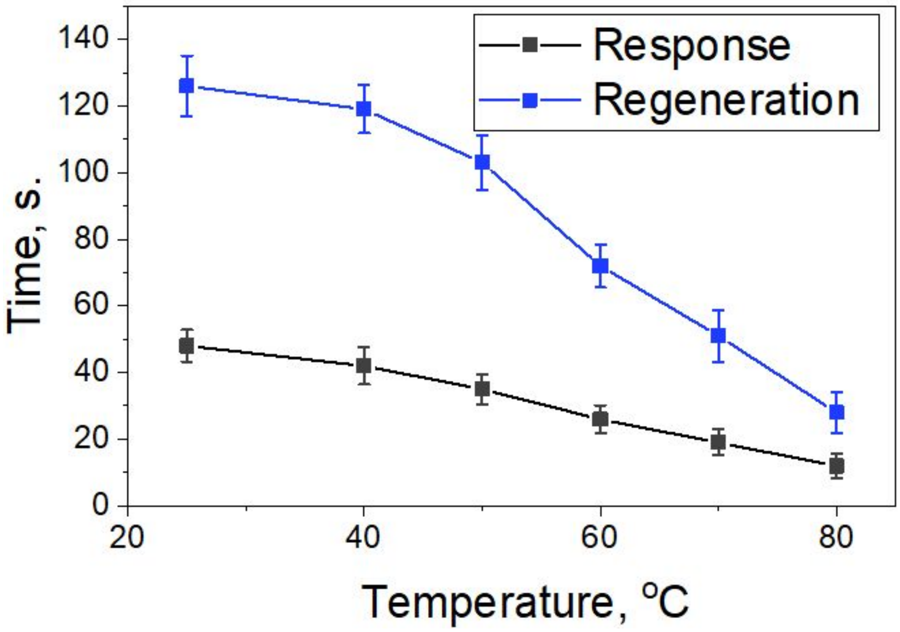

Fig. S15 Time of sensor response and regeneration as a function of temperature (measured as the time of complete shift of the maximum plasmon absorption band at 30 % vol. H_2, RT and zero humidity).

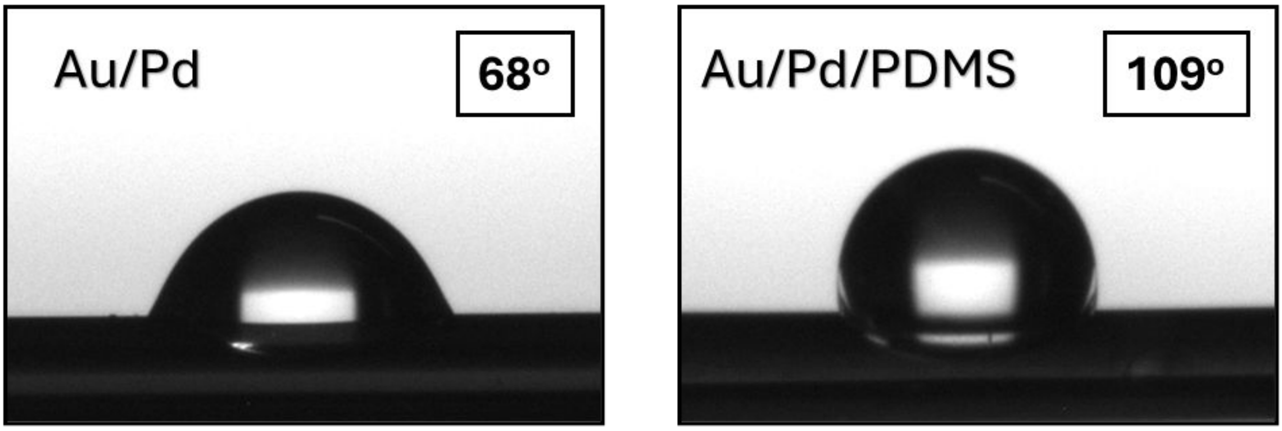

Fig. S16 Water contact angle measured on the PDMS surface vs. Pd surface.

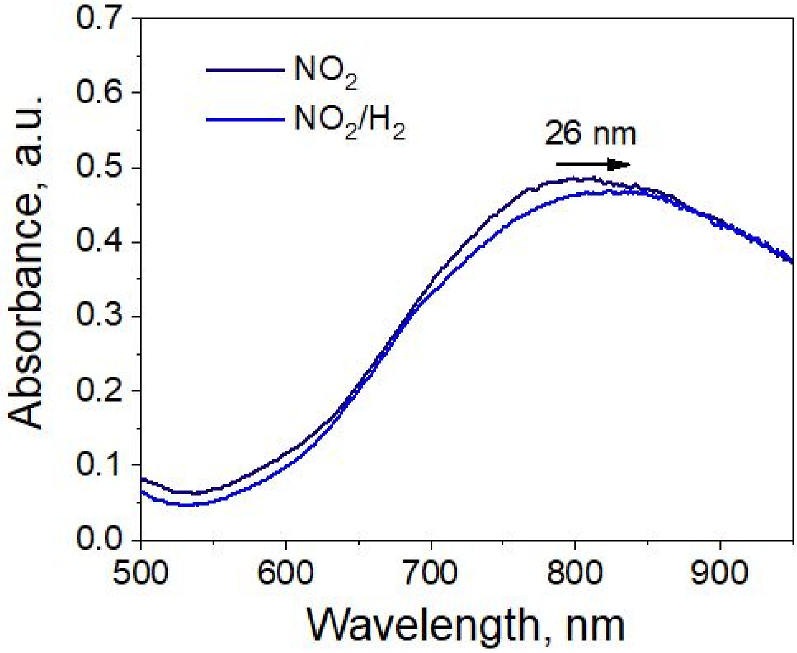

Fig. S17 Sensor response to hydrogen in the mix with NO_2 (30% hydrogen against NO_2 background).

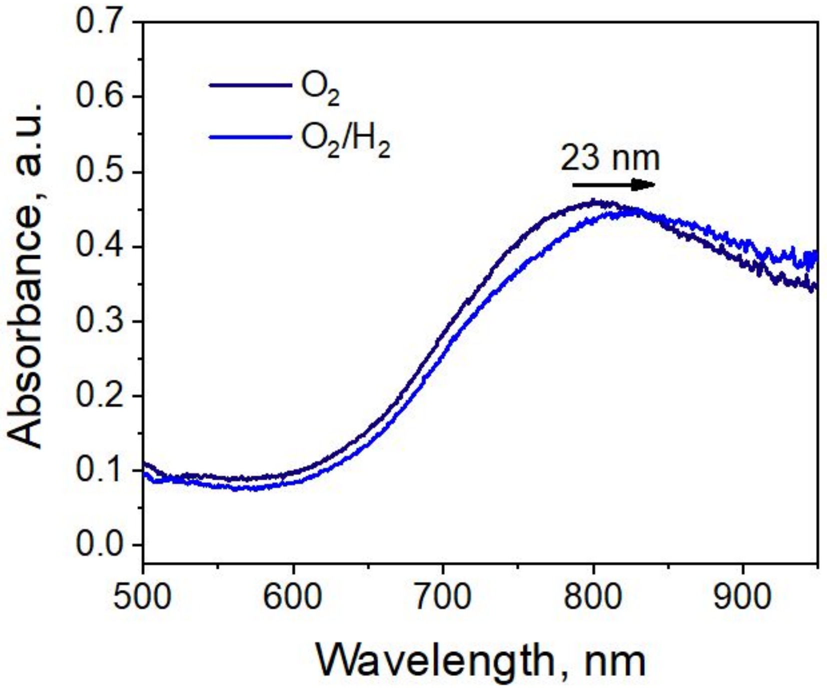

Fig. S18 Sensor response to hydrogen in the mix with O_2 (30% hydrogen against O_2 background).

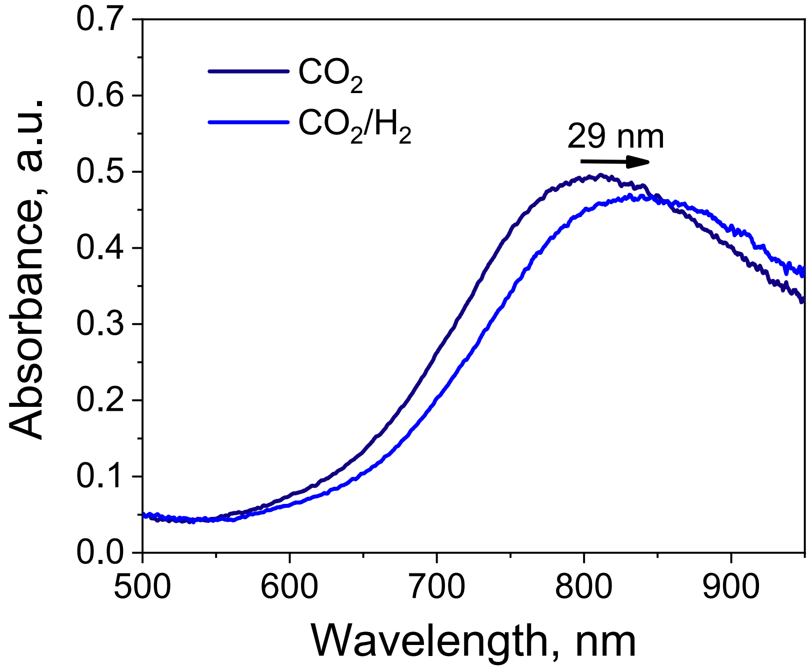

Fig. S19 Sensor response to hydrogen in the mix with CO_2 (30% hydrogen against CO_2 background).

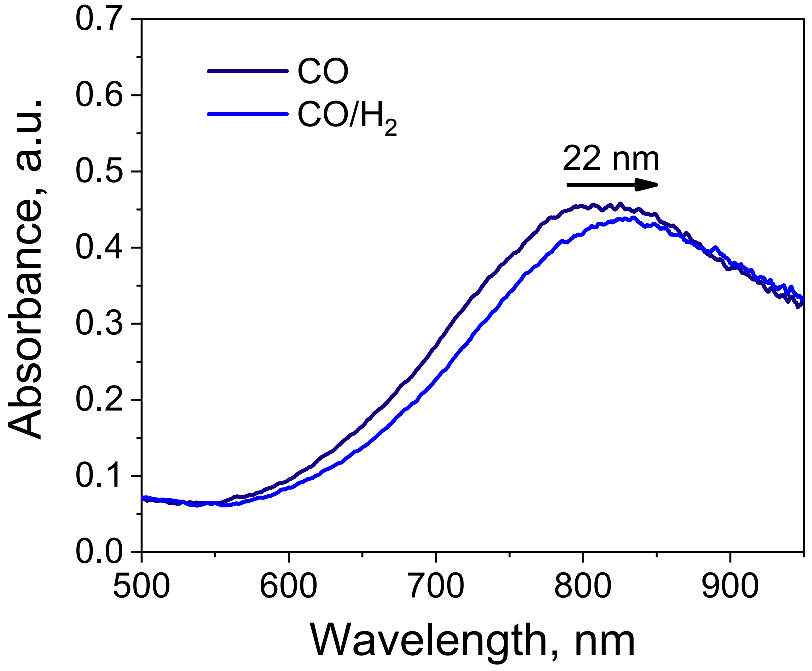

Fig. S20 Sensor response ot hydrogen in the mix with CO (30% hydrogen against CO background).

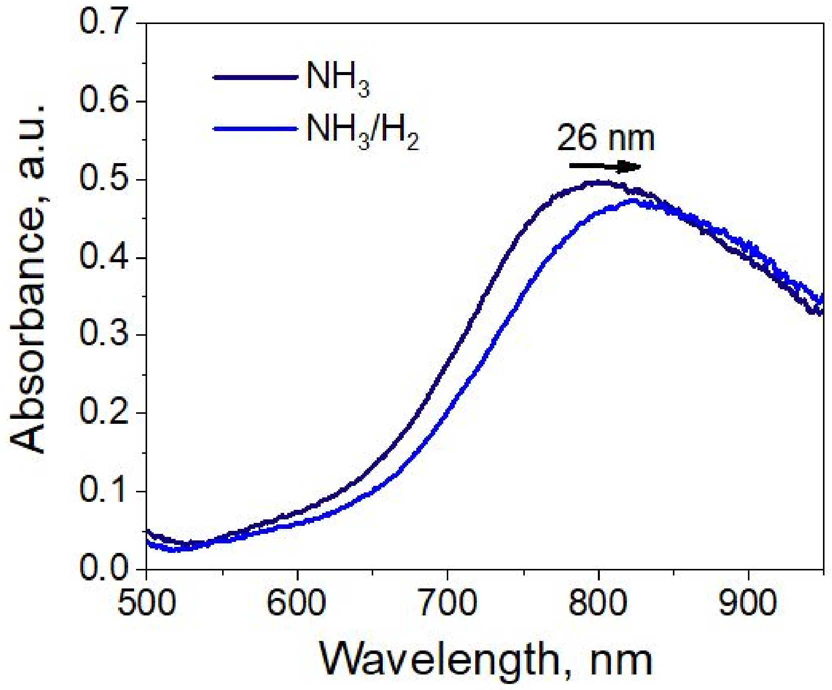

Fig. S21 Sensor response to hydrogen in the mix with NH_3 (30% hydrogen against NH_3 background).

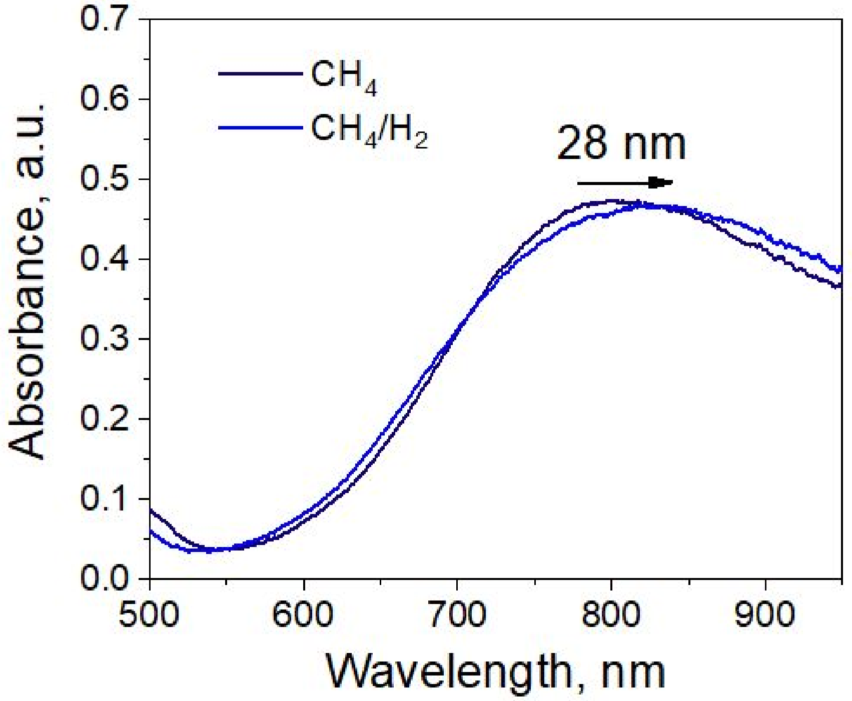

Fig. S22 Sensor response to hydrogen in the mix with CH_4 (30% hydrogen against CH_4 background).

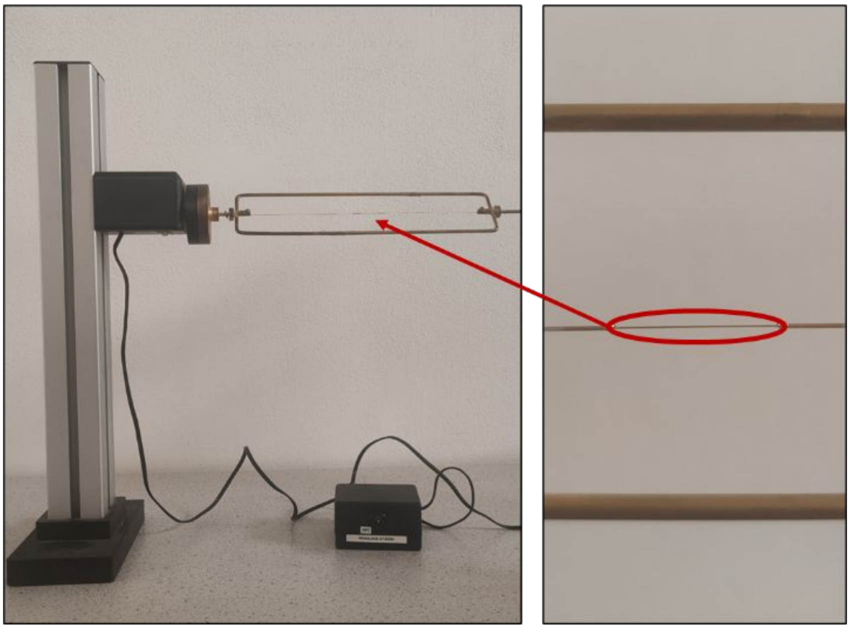

Fig. S23 Photo of the experimental set-up used for the curing of PDMS on the optical fibre surface under continuous rotation.

Table S1 Affiliation of the Raman bands of polymers [S1].

Description	Peak position (cm^{-1})		
	PDMS	PMMA	PS
Si–O–Si	488, 1411	-	-
CH_3	708, 2906, 2965	812, 994, 1453	-
Si–CH_3	615	-	-
C–C	-	603	620, 1001, 1200
C–H	-	-	799, 1033, 1200
C=C	-	--	1601

References

(S1) Infrared and Raman Characteristic Group Frequencies: Tables and Charts. 3rd Ed By George Socrates (The University of West London, Middlesex, U.K.). J. Wiley and Sons: Chichester. 2001. Xviii + 348 Pp. $185.00. ISBN: 0-471-85298-8. J. Am. Chem. Soc. 2002, 124 (8), 1830–1830. https://doi.org/10.1021/ja0153520.
